# A Patient With Small Bowel Obstruction Secondary to an Incarcerated Left Obturator Hernia

**DOI:** 10.7759/cureus.98085

**Published:** 2025-11-29

**Authors:** Kabhisha Gunasekaran, Farah Abdul Aziz

**Affiliations:** 1 Acute Surgical Unit/General Surgery, Sir Charles Gairdner Hospital, Nedlands, AUS; 2 Acute Surgical Unit/General Surgery, Royal Perth Hospital, Perth, AUS; 3 Acute Surgical Unit, Fiona Stanley Hospital, Murdoch, AUS; 4 Breast and General Surgery, Sir Charles Gairdner Hospital, Nedlands, AUS

**Keywords:** elderly patients, frail elderly, obturator hernias, small-bowel obstruction, surgical repair of hernia

## Abstract

Obturator hernia, an infrequent pelvic hernia, which primarily affects thin elderly women, often leads to intestinal obstruction due to diagnosis delay resulting from the absence of specific symptoms and detectable signs. The delay in diagnosis and surgical intervention contributes to its relatively high morbidity and mortality rates. This case report discusses a woman in her 80s with a non-underweight BMI of 19.9 kg/m2 who experienced small bowel obstruction (SBO) due to an incarcerated left obturator hernia, which was found relatively late at day three post symptoms. The patient had a history of rapid unintentional weight loss, reduced oral intake, as well as symptoms suggestive of bowel obstruction, such as vomiting and obstipation, and was diagnosed preoperatively via Computerized Tomography (CT) imaging findings showing an incarcerated left obturator hernia.

This case report presents a unique case of left-sided incarcerated obturator hernia, which is less common compared to right-sided obturator hernia due to anatomical reasons. Surgical intervention involved a mini lower midline laparotomy for the reduction and repair of the obturator hernia, and fortunately, no bowel resection was required due to the viability of bowel intra-operatively despite clinical concern of bowel ischemia due to the delay in surgical intervention resulting from the transfer of the patient from a regional area. Although many cases of obturator hernia are usually diagnosed intra-operatively, in this case report, we highlight the importance of considering the diagnosis of obturator hernia, especially in cases of elderly, thin female patients presenting with bowel obstruction, and to ensure an early pre-operative diagnosis, comprehensive history assessment, meticulous physical examination, and thorough diagnostic procedures, including a CT scan.

## Introduction

Obturator hernia is a rare hernia type that is often not palpable (occult) and accounts for 1% of all hernias [1,2]. It occurs when there is herniation of the hernia sac through the obturator foramen, where the obturator nerves and muscular structures pass through [1]. Diagnosis delay is common due to diagnostic challenges resulting from vague symptoms and findings, particularly in elderly, underweight (BMI ≤18.5 kg/m2) female patients, leading to heightened risk of bowel strangulation, which can lead to perforation, causing substantial morbidity and a high mortality rate [2,3]. Given the diagnostic challenges posed by this condition, CT has emerged as a vital tool for prompt diagnosis, enabling early surgical intervention resulting in diminished morbidity and mortality rates [1,3].

We present a case of a rare left incarcerated obturator hernia. The patient’s history led to our suspicion of small bowel obstruction (SBO), likely secondary to adhesions from previous surgeries. The clinical diagnosis was established preoperatively through CT findings, which showed a loop of bowel between the pectineus and obturator externus muscles. The CT also demonstrated a transition point at the mid-ileal region with preserved bowel enhancement and the presence of free fluid. These findings supported the intraoperative decision to avoid bowel resection and to perform a primary suture repair without mesh due to infection risk. Left-sided obturator hernia has less predominance than right-sided obturator hernia due to sigmoid shielding on the left side, and in this case of a non-underweight patient, it could be attributed to the fact that the patient had rapid weight loss and sarcopenia [2].

The limitation of this case report is that the physical examination specific to obturator hernia was not performed in this patient; however, the clinical findings suggested SBO, which led to the CT diagnosis and supported the intraoperative decision to avoid resection and choose the surgical technique. A mini lower midline laparotomy was preferred over laparoscopy in this case. This approach avoided the risk of failure to reduce the hernia laparoscopically and the potential for bowel perforation due to difficult manipulation with the laparoscopic technique. The delayed presentation and concern for impending bowel ischemia also influenced this decision, as the CT had been performed prior to the patient’s transfer from the regional hospital to the tertiary hospital.

## Case presentation

A female patient in her 80s presented to the Emergency Department (ED) after being transferred from a regional hospital. She had a three-day history of vomiting and obstipation, and CT findings showed an incarcerated obturator hernia. She also had hypokalaemia and ECG changes of ST elevation in leads I, II, and III, which normalized after electrolyte replacement. She was transferred to the tertiary center due to the anticipated need for intensive care unit (ICU) support postoperatively. The patient did not complain of any chest pain. She was recently admitted for two weeks due to reduced oral intake and unintentional weight loss from 65 kg to 51 kg over a four-month duration. Her BMI was 19.9 kg/m2. Her gastroscopy revealed a 4 cm hiatus hernia, short oesophageal stricture in the gastro-oesophageal junction, and severe ulcerative reflux oesophagitis. Her colonoscopy showed descending and sigmoid colon diverticular disease.

She has no past medical history. Her past surgical history includes a hysterectomy and left inguinal hernia repair. She does not consume regular medications and refrains from smoking and alcohol consumption. She has no known family history of malignancy or inflammatory bowel disease. On examination, her vital signs were within normal ranges. Her abdomen was soft, but she experienced tenderness in the left lower quadrant with no guarding or rigidity. There were no masses palpable in either groin area. Her blood test results indicated elevated inflammatory markers, showing a white cell count (WCC) of 11.42 x 10*9/L and a C-reactive protein (CRP) of 54 mg/L. Additionally, she had an electrolyte imbalance showing hypokalaemia, hypomagnesemia, and hypophosphatemia, which were appropriately replaced. Her lactate level was 1 mmol/L. Table 1 illustrates the laboratory findings of this patient [4].

**Table 1:** Laboratory findings of the patient with normal range for reference [4]

	Blood count level	Repeat/Post-correction level pre-operation	Normal range
Haemoglobin (Hb)	100 g/L	99 g/L	115-160 g/L
White cell count (WCC)	11.42 10*9/L	10.71 10*9/L	4.00-11.00 10*9/L
Platelet (Plt)	262 10*9/L	265 10*9/L	150-400 10*9/L
C-reactive protein (CRP)	54 mg/L	58 mg/L	<5.0 mg/L
Creatinine (Cr)	36 umol/L	37 umol/L	45-90 umol/L
Estimated glomerular filtration rate (eGFR)	>90 mL/min/1.73m2	>90 mL/min/1.73m2	>60 mL/min/1.73m2
Potassium	2.4 mmol/L	2.8 mmol/L	3.5-5.2 mmol/L
Magnesium	0.55 mmol/L	0.98 mmol/L (5 hours post-surgery)	0.70-1.10 mmol/L
Phosphate	0.46 mmol/L	0.76 mmol/L (1 day post surgery)	0.75-1.50 mmol/L
Lactate	1 mmol/L	0.7 mmol/L	<2.0 mmol/L
Albumin (Alb)	29 g/L	28 g/L	35-50 g/L
Troponin I (TnI)	12 ng/L	17 ng/L	<16 ng/L

**Figure 1:**
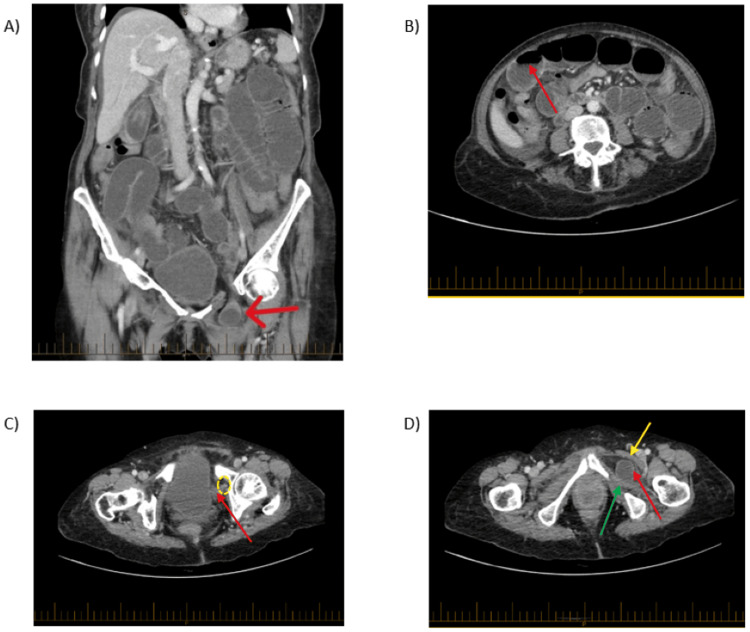
CT slice images of abdomen/pelvis with intravenous contrast in portal venous phase seen on window (W):369 level (L):40 level. A: CT coronal slice image of 5 mm slice thickness demonstrating small bowel obstruction (SBO) with dilatation of the small bowel with mesenteric stranding secondary to a left obturator hernia (red arrow) as the transition point where there is a protruding short segment of mid-ileum. There is a small volume of free fluid in the abdomen and pelvis, most notably surrounding the right lobe of the liver, with no evidence of portal venous gas or intra-abdominal free air. B: CT axial slice image of 3 mm slice thickness showing dilated loops of small bowel measuring up to 4 cm in diameter associated with air fluid levels (red arrow) with no evidence of abnormal mural thickening of bowel loops or pneumatosis intestinalis. C: CT axial slice image of 3 mm slice thickness showing an obturator canal (yellow dotted oval) and hernia protruding into the obturator canal (red arrow). D: CT axial slice image of 3 mm slice thickness showing loop of bowel (red arrow) between the pectineus (yellow arrow) and obturator externus (green arrow) [5].

She had a CT scan performed on day three following obstruction symptoms to identify the transition point, as there was suspicion of bowel obstruction, which led to the clinical diagnosis. The differential diagnoses for this patient were SBO secondary to adhesions resulting from her previous surgeries, diverticulitis, and colitis. She had a CT scan performed to identify the transition point, as there was high suspicion of small bowel obstruction based on her clinical history, which led to the diagnosis of left obturator hernia. The CT scan (Figure 1) demonstrated SBO secondary to a left obturator hernia, with the transition point identified at the mid-ileum [5]. The imaging also showed a small volume of free fluid in the abdomen and pelvis, although there was no evidence of free gas indicative of bowel perforation. She arrived at the tertiary centre ED 20 hours after CT scan.

She was kept nil by mouth with intravenous fluid and a nasogastric (NG) tube, and an indwelling catheter (IDC) was inserted prior to transfer to the tertiary centre to carefully monitor her fluid balance. She received sufficient analgesia and antiemetic medications as well as proton pump inhibitors and anti-coagulation. She was American Society of Anesthesiologists (ASA) III, and her repeat ECG preoperatively showed sinus tachycardia of 122 bpm with left axis deviation and no ST elevation. Following informed consent, she underwent a lower midline laparotomy with reduction and repair of the left obturator hernia after two hours of arrival at the tertiary centre ED. The surgical findings were SBO due to the left obturator hernia as well as the presence of free fluid, along with distended bowel loops proximal to the obstruction site and collapsed loops of bowel distal to it. A Richter hernia involving a small bowel loop in the left obturator defect was successfully reduced, and the hernia defect was closed with 3/0 polydioxanone suture (PDS), and the peritoneum layer was opposed over the left obturator foramen with 3/0 PDS. The appearance of the bowel was healthy and viable, with a shiny surface and active peristalsis observed. Consequently, there was no necessity for bowel resection.

Following surgery, she experienced an ongoing postoperative Clavien-Dindo Grade 2 ileus, which was managed by providing parenteral nutrition on day four post-surgery, and her ileus resolved on day seven post-surgery. She was able to tolerate a high volume of clear fluid orally with minimal NGoutput on day one post-surgery, but then experienced increasing volume of NG output since day two post-surgery. She was not able to pass flatus and open bowels up to day seven post-surgery. Therefore, peripheral parenteral nutrition (PPN) was started on day four post-surgery and converted to total parenteral nutrition (TPN) on day seven post-surgery, which was ceased on day 11 post-surgery. An abdominal X-ray (AXR) six hours following diatrizoate administration was performed on day nine post-surgery, which revealed dilated small bowel loops containing diatrizoate, measuring up to 44 mm in cross-sectional diameter, with some contrast observed in the right colon, where she then had a large amount of bowel opening [5]. Her diet was upgraded to a nourishing fluid and soft diet on day nine and day ten, respectively, post-surgery. The NG was spigotted on day nine post-surgery and subsequently removed on day 11 post-surgery. She was able to continue a normal diet with no nausea or vomiting on day 12 post-surgery.

She continued to progress well; however, due to a delay in returning to her baseline in terms of mobility and function, she was offered rehabilitation after discharge, which was two weeks post-surgery. She was also referred to an outpatient cardiology clinic as a non-urgent referral with a transthoracic echocardiogram as a follow-up for the ECG changes found preoperatively to investigate for any underlying coronary lesions. At her clinic review six weeks after the surgery, her progress appeared positive following rehabilitation with no hernia recurrence, leading to her discharge from the General Surgery Outpatient Clinic.

## Discussion

The earliest documentation of obturator hernia dates back to 1724 by Ronsil [1,3]. The incidence of obturator hernia is relatively rare, accounting for approximately 1% of hernias and 0.2-1.6% of cases involving SBO [1,3,6,7]. Among various abdominal wall hernias, the obturator hernia has the highest reported mortality rate, ranging from 13% to 40% [6,7]. As the time to surgery increases, the risk of bowel resection increases as well, thus increasing morbidity and mortality. Obturator hernia is six to nine times more likely in underweight and multiparous women aged 70 and above compared to men [2]. This case report is unique as the patient had a non-underweight BMI.

**Figure 2:**
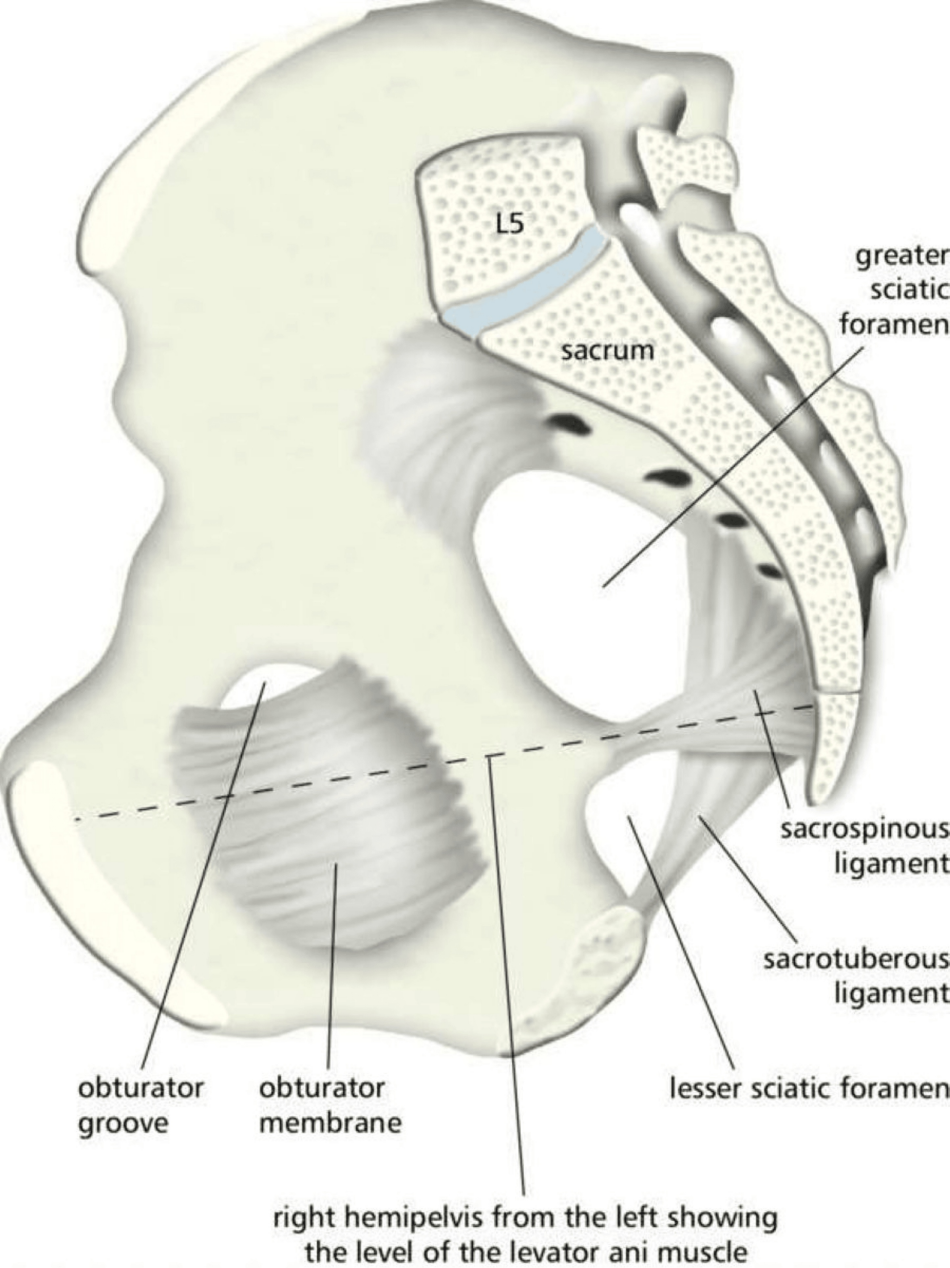
Hemipelvis and pelvic ligaments Shows the obturator foramen containing the obturator canal (obturator groove) at the superior end, where the obturator hernia contents herniate through. Source: Royal College of Surgeons of Ireland, licensed under CC BY-NC-SA 1.0 [10].

This type of hernia arises due to the loss of protective preperitoneal fat and lymphatic tissue (corpus adiposum) around the obturator canal, which normally accommodates the obturator nerve and muscle. This canal, measuring 2-3 cm in length and about 1 cm in width, allows the hernia sac to pass through, as shown in Figure 2 [2,3,7-10]. Situated beneath the pelvic acetabulum, the obturator foramen contains the obturator nerve, artery, and vein, enveloped by a cushion of fat. Its boundaries are defined by the superior pubic ramus superiorly and the ramus of the ischium inferiorly [2].

Obturator hernia primarily affects multiparous and elderly emaciated women aged 70 to 90 due to the presence of a wider pelvis as a result of pelvic peritoneum relaxation following childbirth, as well as a larger and horizontal obturator canal contributing to the nickname, "little old lady's hernia” [1,3,6,11,12]. Several comorbidities, including chronic obstructive pulmonary disease, malnutrition, chronic constipation, ascites, and conditions raising intra-abdominal pressure, are considered risk factors for developing obturator hernia [1,3,12]. In most cases, the hernial sac contains the small intestine, with the Richter type being the most common presentation seen in 41-100% of cases, causing partial intestinal obstruction as shown in this case report. Less frequently, other contents like the large intestine, appendix, omentum, bladder, uterus, fallopian tube, and ovaries can be involved [8,13,14].

The condition is three times more common on the right side, as the sigmoid colon usually offers protection to the obturator canal on the left side [2,6,11]. Bilateral hernias are encountered in about 6% of cases [13]. This case report presents an atypical occurrence as the hernia manifested on the left side. The incidence of left-sided obturator hernia in this patient, despite sigmoid shielding, could be secondary to rapid weight loss, sarcopenia, and prior pelvic region surgery, such as hysterectomy and ipsilateral inguinal repair.

Obturator hernia evolves through three stages: the initial stage involves preperitoneal tissue entering the pelvic opening of the obturator canal, followed by the formation of a dimple in the peritoneum, leading to the creation of a peritoneal sac covering the obturator canal. In the third stage, symptoms arise due to visceral herniation into the peritoneal sac [15].

Patients typically present with acute intestinal obstruction [1,3,7,13]. Various clinical signs suggest obturator hernia [2]. The positive Howship-Romberg sign, arising from intermittent irritation of the anterior branch of the obturator nerve, was reported in 13-65% of obturator hernia cases [3,16]. In 1840, John Howship and in 1845, Heinrich Romberg elucidated that the cardinal signs of obturator hernia involve acute pain due to the obturator nerve incarceration along the inner side of the thigh, which is exacerbated by extending, abducting, and medially rotating the thigh [3]. The Hannington-Kiff sign, which offers higher specificity than the pathognomonic Howship-Romberg sign, is less frequently observed, keeping in mind that the clinical findings are examiner-dependent [2,11].

The Hannington-Kiff sign is detected by an absence of thigh adductor reflex due to the compression of the obturator nerve, which is shown by a lack of contraction of the adductor muscle when it is percussed 5 cm above the knee [2]. Clinical suspicion of obturator hernia is further heightened when a palpable mass is felt during per-rectal and per-vaginal examinations, which were not performed in this patient [7]. The four cardinal signs in patients with obturator hernia consist of nonspecific and vague signs and symptoms, including dull and crampy abdominal pain, nausea and vomiting, the occurrence of pain along the medial aspect of the thigh known as Howship-Romberg's sign, and recurrent episodes of intestinal obstruction or palpable mass on the medial aspect of the thigh [7,12].

Early diagnosis of obturator hernia is challenging due to its nonspecific symptoms and signs. Various imaging techniques aid in the diagnosis of obturator hernia, including ultrasonography and CT scan. Contrast-Enhanced Computed Tomography (CECT) has emerged as the mainstay for early and accurate diagnosis of obturator hernia [13]. CT scan has the highest sensitivity and accuracy for obturator hernia, with a diagnostic value ranging from 78% to 100%, generally estimated at 90% [1,3,14]. Plain AXR films may show features of gas in the obturator site, suggesting a hernia [15].

In this case report, CT imaging validated the preoperative diagnosis of obturator hernia, allowing for prompt surgical intervention, thus obviating the need for bowel resection, as the loop of bowel between the pectineus and obturator externus with mid-ileal transition point showed preserved enhancement, and no features of closed-loop bowel obstruction or free air. Open low midline was preferred in this case due to possible impending bowel ischemia secondary to delayed presentation and in an effort to reduce the risk of difficult reduction, which may lead to potential bowel perforation. No mesh was used in this case as there were concerns of mesh infection due to contamination, as suggested by the presence of free fluid intraoperatively. The obturator foramen defect was closed and layered by a peritoneum layer using 3/0 PDS. The herniated bowel was deemed viable due to a healthy appearance with a shiny surface and active peristalsis. It is important to inspect the contralateral obturator canal as well intraoperatively, as there are chances of bilateral obturator hernia occurring.

Studies emphasize that the duration of symptoms significantly influences the likelihood of bowel resection. Thus, expedited diagnosis within hours rather than days is crucial [1]. Delayed diagnosis, on the other hand, leads to bowel ischemia and an elevated risk of perforation, resulting in high morbidity and a post-operative mortality rate as high as 70% [2,3]. In this case report, the patient was diagnosed with an incarcerated obturator hernia three days after symptoms; however, fortunately, it did not lead to bowel ischemia. Factors contributing to increased mortality among patients with obturator hernia encompass poor physiological reserves, medical comorbidities, higher rate of intestinal strangulation, as well as diagnostic and management delays due to non-specific signs and symptoms, which subsequently lead to bowel necrosis and perforation [2,8]. Bowel resection and postoperative complications are the important factors leading to postoperative mortality [14].

The first successful repair of obturator hernia was achieved by Henry Obre in 1851 [7]. The sole treatment for obturator hernia is surgical intervention, through various approaches including inguinal, retropubic, and transperitoneal techniques. A midline infra-umbilical vertical approach is preferred for acute presentation, while other methods can be applied if the hernia is diagnosed preoperatively in a non-acute setting [11]. In this case, a laparotomy via a low midline incision was applied due to the advantage of better exposure of the obturator ring, avoidance of obturator vessels, and facilitation of bowel inspection and potential resection if required [1,6,7,12]. Laparoscopic repair - using either the transabdominal preperitoneal or extraperitoneal approach - is another option for diagnosing, reducing, and repairing obturator hernia. It is particularly suitable for non-strangulated hernias, unless contraindicated by infection or peritonitis, where the technique becomes more challenging and the operative time is longer [1,2].

The laparoscopy approach has advantages of reduced post-operative pain, decreased ileus, shorter hospital stays, and lower overall complications [1,2,7,11]. Laparoscopy approach provides optimal visualisation of the pelvis and may be safely conducted in high-risk patients if done cautiously [17]. As surgeons are now mastering laparoscopic and robotic surgery, many hernia surgeries are now commonly being managed with the minimally invasive approach [9]. A mini lower midline laparotomy was selected over laparoscopy to avoid the risk of unsuccessful hernia reduction and the potential for bowel perforation from difficult laparoscopic manipulation. This decision was influenced by the delayed presentation and the concern for impending bowel ischemia, as the CT scan had been performed before the patient was transferred from the regional hospital to the tertiary center.

**Figure 3:**
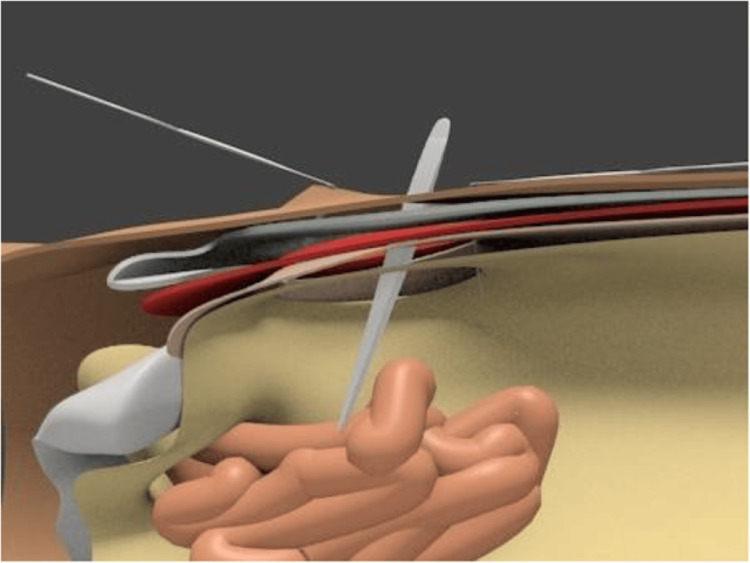
A simple schematic diagram showing the obturator hernia containing small bowel passing through the obturator foramen defect Source: Hosoi et al. [18]. Licensed under the Creative Commons CC BY 4.0 license.

**Figure 4:**
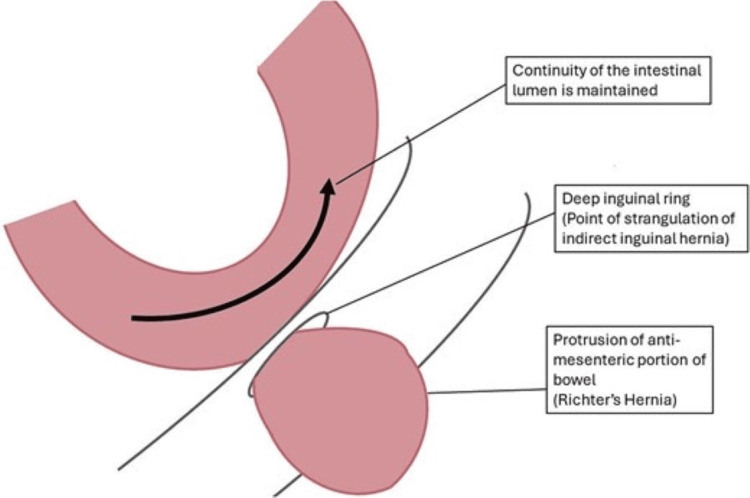
A diagram showing Richter-type inguinal hernia Source: Smith et al. [19]. Licensed under the Creative Commons CC BY 4.0 license.

**Figure 5:**
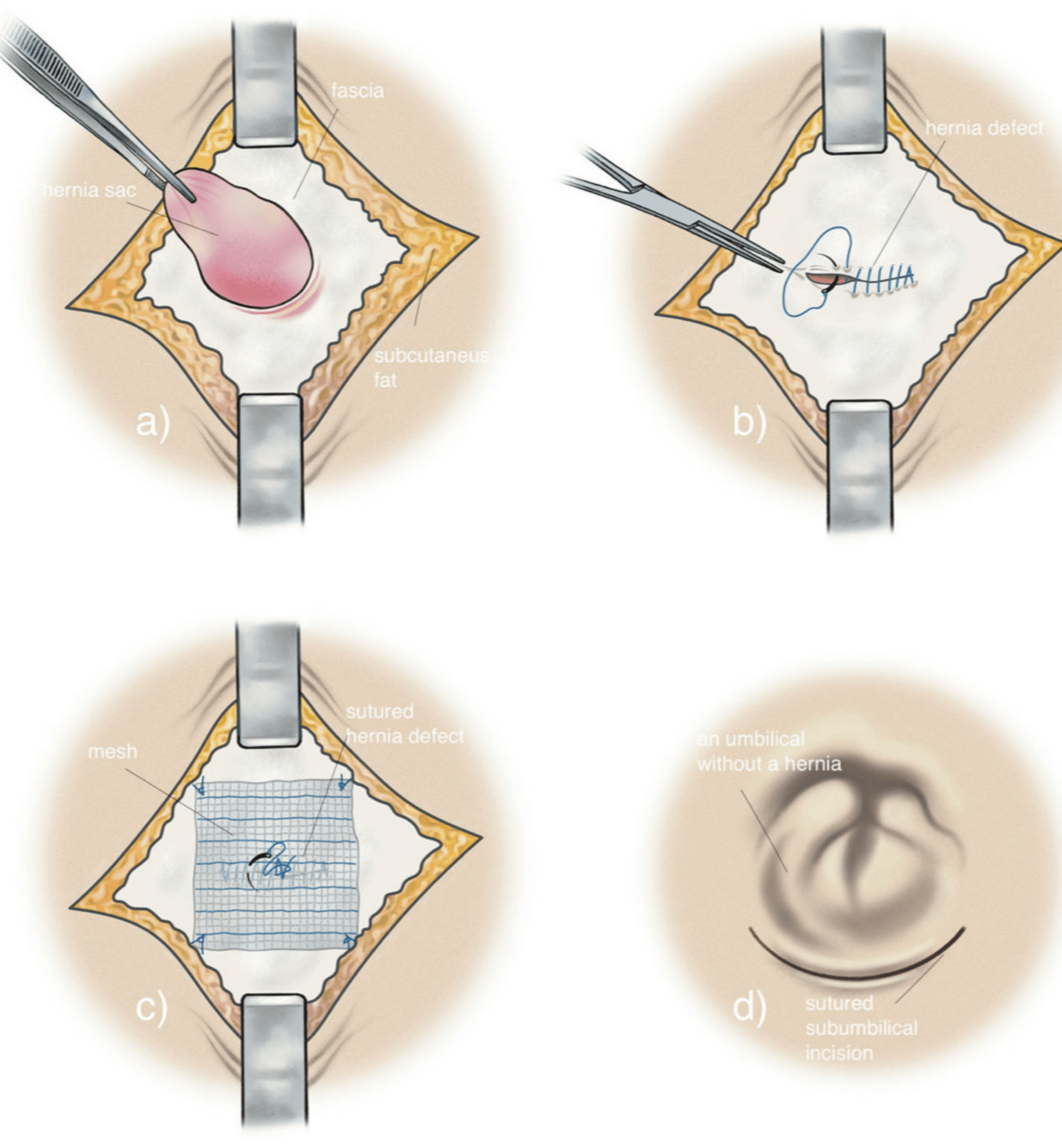
Primary suture closure repair technique with mesh of umbilical hernia Source: Melkemichel et al. [20]. Licensed under the Creative Commons CC BY 4.0 license.

Methods of repair and closure encompass simple suturing, obturator closure with adjacent tissues, and mesh placement [1,3,7]. The preferred technique typically involves simple closure of the hernial defect using interrupted sutures, a method chosen in this case due to its low recurrence rate of less than 10% [1,7]. Primary closure without the utilization of prosthetic materials is advisable when there is a high risk of infection [14]. For larger obturator hernias, mesh reinforcement may be necessary [8]. Figures 3-5 show a simple schematic diagram illustrating the obturator defect, Richter hernia, and primary suture closure of a hernia defect with mesh [18,19,20]. Overall recurrence rate at 3 years post-surgery has been noted to range from 5.1% to 7.1% [14].

Summary of recent cases

A summary of the recent left obturator hernia cases has been presented in Table 1 [2,6,9,15,17].

**Table 2:** Summary of recent left obturator hernia cases

Case	#1 Diab et al. [2]	#2 Devarakonda et al. [6]	#3 Delgado et al. [9]	#4 Aryal et al. [15]	#5 Petrushnko et al. [17]
Incidence	2021	2020	2022	2023	2019
Age (years)/ gender	83/Female	81/Female	89/Female	70/Female	79/Female
Duration of symptoms (days)	1	4	6	3	Not available
Intestinal obstruction	Yes	Yes	Yes	Yes	Yes
Previous abdominal operation	No	Yes	Yes	No	No
Howship-Romberg sign	Absent	Not performed	Not performed	Not performed	Not available
CT (abdomen/ pelvis) pre-operative	Yes	Yes	Yes	Yes	Yes
Preoperative diagnosis of obturator hernia	Yes	Yes	Yes	Yes	Yes
Hernia content	Small bowel	Small bowel	Small bowel	Small bowel	Small bowel
Operative approach	Laparoscopy	Laparotomy	Laparotomy	Laparotomy	Laparoscopic totally extra-peritoneal (TEP) repair
Repair type	Not available	Interrupted Prolene sutures	Woven polypropylene mesh + 2-0 Prolene suture	Not available	Self-gripping mesh (Progrip)
Bowel resection	No	No	No	No	No
Recurrence	Not available	No after 2 years	No after 30 days	Not available	Not available

Limitations

The limitations of this case report are: it discusses a single case, has a short follow-up duration of six weeks, and has missing intraoperative images. The operation report also does not mention the defect size, defect edge quality, Richter segment length, or description of contralateral canal inspection.

Take-home points

Left-sided incarcerated obturator hernia is a rare diagnosis that needs to be considered in an elderly patient with rapid weight loss presenting with bowel-obstruction symptoms.

CT-guided viability assessment of the obturator hernia content is crucial to guide the selection of the most pragmatic surgical intervention.

It is important to inspect the contralateral site of the obturator foramen in a systematic manner intraoperatively due to the chances of bilateral obturator hernia occurring.

## Conclusions

It is important to consider the rare diagnosis of incarcerated obturator hernia in elderly, frail women who present with bowel obstruction symptoms. Prompt diagnosis of incarcerated obturator hernia derived from obtaining a thorough history of abdominal pain, nausea, vomiting, and recurrent bowel obstructions, as well as physical examination to elicit evidence of obturator hernia, is crucial. Early CT scan as the radiological method of choice is useful for the diagnosis of incarcerated obturator hernia, leading to early interventions, which help in reducing morbidity and mortality. This case describes a left-sided obturator hernia in an elderly female with a BMI ≥18.5 kg/m² and recent rapid weight loss, in whom CT-guided assessment of bowel viability helped avoid resection. A low or infra-umbilical midline laparotomy for the incarcerated obturator hernia, as described in this case report, is beneficial in an acute setting, as it is the safest and fastest approach in facilitating bowel exploration and resection, if required. An open primary suture repair with no mesh was performed in this case due to the risk of infection secondary to the presence of free fluid in the abdomen and pelvis.

## References

[1] Cai X, Song X, Cai X (2012). Strangulated intestinal obstruction secondary to a typical obturator hernia: a case report with literature review. Int J Med Sci.

[2] Diab J, Badiani S, Di Re A, Berney CR (2021). An elderly woman's limp: obturator hernia as a rare cause of small bowel obstruction. Aust J Gen Pract.

[3] Kisaoglu A, Ozogul B, Yuce I, Bayramoglu A, Atamanalp SS (2014). Obturator hernia, a rare cause of small bowel obstruction: case report. Eurasian J Med.

[4] (2025). i.Clinical Manager - Clinical Management System (CMS) - Dedalus NE. https://www.dedalus.com/ne/en/our-offer/products/clinical-management-system-cms/.

[5] (2025). InteleConnect EV portal frequently asked questions (FAQs). https://sjra.com/wp-content/uploads/2024/08/InteleConnect-EV-Portal-Reference-Guide.pdf.

[6] Devarakonda A, Gupta-Kaistha A, Kulkarni N (2020). Small bowel obstruction secondary to obturator hernia in an elderly female. J Surg Case Rep.

[7] Mantoo SK, Mak K, Tan TJ (2009). Obturator hernia: diagnosis and treatment in the modern era. Sing Med J.

[8] Servis D, Miočinović M, Patrlj L (2012). Richter type of incarcerated obturator hernia: misleading all the way. Acta Clin Croat.

[9] Delgado A, Bhuller SB, Phan P, Weaver J (2022). Rare case of obturator hernia: surgical anatomy, planning, and considerations. SAGE Open Med Case Rep.

[10] (2025). RCSI - Drawing hemipelvis and pelvic ligaments - English labels | AnatomyTOOL. Health Education Assets Library. University of Utah.

[11] Shreshtha S (2016). Obturator hernia: an uncommon cause of small bowel obstruction. J Postgrad Med.

[12] Kulkarni SR, Punamiya AR, Naniwadekar RG, Janugade HB, Chotai TD, Vimal Singh T, Natchair A (2013). Obturator hernia: a diagnostic challenge. Int J Surg Case Rep.

[13] Suresh A, Chinnakkulam Kandhasamy S, Sahoo AK, Amaranathan A, Vishnu Prasad NR (2018). A masquerading and unconventional cause of dynamic intestinal obstruction: strangulated obturator hernia. Cureus.

[14] Li Z, Gu C, Wei M, Yuan X, Wang Z (2021). Diagnosis and treatment of obturator hernia: retrospective analysis of 86 clinical cases at a single institution. BMC Surg.

[15] Aryal M, Keshari S, Pandey A, Pandey A, Paudel I (2023). A rare case of left-sided obturator hernia diagnosed by computed tomography. Clin Case Rep.

[16] Aydin I, Yucel AF, Pergel A, Sahin DA (2013). Obturator hernia: a rare case of acute mechanical intestinal obstruction. Case Rep Surg.

[17] Petrushnko W, Isaacs A, Hackland T, Ghusn M (2019). Case report: laparoscopic totally extraperitoneal repair of an obturator hernia with self-gripping mesh under spinal anaesthesia. Int J Surg Case Rep.

[18] Hosoi Y, Asano H, Fukano H, Shinozuka N (2020). Treatment outcomes of Kugel repair for obturator hernias: a retrospective study. BMC Surg.

[19] Smith CR, Chatzikonstantinou M (2024). Early surgical intervention is critical for strangulated Richter's hernia. J Surg Case Rep.

[20] Melkemichel M, Bringman S, Granåsen G, Widhe B (2021). SUMMER trial: mesh versus suture repair in small umbilical hernias in adults-a study protocol for a prospective randomized double-blind multicenter clinical trial. Trials.

